# Supplementary material for: M-ReDet: A mamba-based method for remote sensing ship object detection and fine-grained recognition
Source: PLoS One. 2025 Aug 21;20(8):e0330485. doi: 10.1371/journal.pone.0330485 (PMC12370123; doi:10.1371/journal.pone.0330485)
Supplement: Attachment — Submitted filename: Comments.pdf [file pone.0330485.s001.pdf]

1. Abstract
    - In addition to emphasizing that ship detection in remote sensing imagery is currently a hot topic, it would be beneficial to specify the application domains in which such detection tasks are conducted. Doing so would help the reader better understand the practical relevance of the research question and potentially reveal its target users or beneficiaries.
    - Please specify the image format (RGB,Multispectral,etc.) of the dataset used in the study within the abstract. This information will enhance the clarity and reproducibility of the work.
2. Introduction
    - Please clarify the novelty of the study more explicitly at the end of the Introduction section. Clearly stating the unique contributions will help distinguish this work from existing literature.
3. Related work
    - The content of the Related Work section is expected to align with the statement in your sentence: "The commonly used remote sensing object detection algorithms can be categorized into three classes: the Convolutional Neural Network, the Transformer [1], and the Mamba...". Ensuring coherence between this categorization and the subsequent discussion will improve the clarity and structure of the manuscript.

    3.1.1. ReDet

    The section is appropriate

    3.1.2. Attention mechanisms

    The section is appropriate

    3.1.3. Mamba

    The section is appropriate
4. Our Work

    4.1.1. M-ReDet

    The section is appropriate

    4.1.2. SOPM

    The section is appropriate

    4.1.3. SFRM

    The section is appropriate

4.1.4. Optimizing the loss function

- Under the heading "Optimizing the Loss Function," merely changing the loss function appears inconsistent. Readers may expect an actual optimization method or strategy within this section. It is advisable to either revise the heading to better reflect the content or expand the section to include optimization techniques.
- In addition to KFIoU Loss, it is recommended to include a information about Focal Loss as well.

5. Experiment and Result Analysis

5.1.1. Experimental environment and parameter configuration

- Presenting the details under the "Experimental Environment and Parameter Configuration" section in a tabular format would enhance readability and clarity for the reader. Additionally, it is recommended to include resource consumption metrics of your developed model compared to the baseline model, such as RAM usage and training time per epoch. These details will provide a more comprehensive evaluation of the model's efficiency.

5.1.2. Datasets

- Please provide detailed visualizations of the distribution within the datasets using appropriate graphs. This will help readers better understand the data composition and support the validity of the experimental results.

5.1.3. Experimental evaluation indicators

The section is appropriate

5.1.4. Analysis of experimental results

The section is appropriate

5.1.5. Ablation experiments

The section is appropriate

6. Discussion

- In the Discussion section, it would be beneficial to mention potential future work, such as leveraging SAR imagery for improved performance and addressing enhancements to cope with adverse weather conditions. Including these aspects could strengthen the manuscript by highlighting promising directions for further research.

7. Conclusion

   The section is appropriate
