# Supplementary material for: M-ReDet: A mamba-based method for remote sensing ship object detection and fine-grained recognition
Source: PLoS One. 2025 Aug 21;20(8):e0330485. doi: 10.1371/journal.pone.0330485 (PMC12370123; doi:10.1371/journal.pone.0330485)
Supplement: Attachment — Submitted filename: Response to Reviewers.pdf [file pone.0330485.s002.pdf]

## Response to the Reviewers' Comments

Thank you very much for giving us the opportunity to improve the manuscript. The authors are grateful for the reviewers' valuable comments, which have helped us to understand the underlying approach in depth and improved the original manuscript. We think carefully about comments raised by the reviewers and provide an item-by-item list to explain how these comments were addressed.

In the 'Revised Manuscript with Track Changes' file, we use the highlighting to mark the modifications. Point-to-point responses to reviewers' comments are below. Reviewers' comments are in italics and blue, immediately followed by our response.

## Reviewer: 1

**Comments to the Author:**

*The work is well organised and successfully presented. The following paper sets out a proposal for a new detection algorithm with regard to the identification of ships. It is hypothesised that the study will achieve a higher level of success if the following recommendations are taken into consideration during the process of its update.*

**Response：** Thank you very much for your careful review and recognition of our manuscript. We will make cautious revisions based on your feedback. Thank you very much.

**Specific comments:**

*1. It is important to note that all tables and graphs in the study were prepared with the mAP metric exclusively. The application of additional overlap metrics is also recommended. It is recommended that metrics such as DSC, IoU,RAVD and HD95 be deployed.*

**Response：** Thank you very much for your valuable feedback. The mAP is critical in remote sensing object detection tasks. Additionally, the evaluation metrics in the manuscript also utilize AP and Recall. In the loss function, we also use IoU as the training basis for the algorithm. The DSC and other metrics you mentioned are used mainly in the field of image segmentation. Thank you again for your valuable feedback.

*2. It is imperative that references are updated and expanded.*

**Response：** Thank you very much for raising the questions in the references. We have revised the format of the references and added some references.

*3.It is recommended that the resolution quality of the images is increased, with a view to enhancing their clearness and legibility.*

**Response：** Thank you very much for your valuable feedback. Our minimum image resolution is 1024 × 1024, and the image displayed in the PDF file should be a resized version of the original image. You can download and view the original image in the system. Thank you very much for your efforts and recognition.

## Reviewer: 2

**Comments to the Author:**

*I appreciate the effort put into this manuscript. Addressing the suggested revisions will significantly enhance the clarity and impact of your work. By refining these sections, the manuscript will provide greater value to the research community and result in a more robust and informative publication.*

**Response:** Thank you very much for giving us the opportunity to improve the manuscript and pointing out the shortcomings in the manuscript. In this iteration, we have revised the manuscript on the following issues.

**Specific comments:**

*1. Abstract: In addition to emphasizing that ship detection in remote sensing imagery is currently a hot topic, it would be beneficial to specify the application domains in which such detection tasks are conducted. Doing so would help the reader better understand the practical relevance of the research question and potentially reveal its target users or beneficiaries. Please specify the image format (RGB,Multispectral,etc.) of the dataset used in the study within the abstract. This information will enhance the clarity and reproducibility of the work.*

**Response:** Thank you very much for your valuable feedback. Adding application areas and image formats to the abstract can indeed improve the clarity of the research. We have added relevant content.

*2. Introduction: Please clarify the novelty of the study more explicitly at the end of the Introduction section. Clearly stating the unique contributions will help distinguish this work from existing literature.*

**Response:** Thank you for your efforts during the review process. We have made revisions to the work description and innovative design at the end of the manuscript introduction section.

*3. Related work: The content of the Related Work section is expected to align with the statement in your sentence: "The commonly used remote sensing object detection algorithms can be categorized into three classes: the Convolutional Neural Network, the Transformer [1], and the Mamba...". Ensuring coherence between this categorization and the subsequent discussion will improve the clarity and structure of the manuscript.*

**Response:** Thank you very much for your careful review. We have revised the titles of the relevant work sections to align them with the descriptions in the introduction section of the manuscript.

*4. Our Work: Under the heading "Optimizing the Loss Function," merely changing the loss function appears inconsistent. Readers may expect an actual optimization method or strategy within this section. It is advisable to either revise the heading to better reflect the content or expand the section to include optimization techniques. In addition to KFIoU Loss, it is recommended to include a information about Focal Loss as well.*

**Response:** We are very grateful for your valuable suggestions. We have revised the title "Optimizing the Loss Function" to "Changing the Loss Function" to avoid confusion for readers. The manuscript also includes a description of Focal loss.

*5. Experiment and Result Analysis: Presenting the details under the "Experimental Environment and Parameter Configuration" section in a tabular format would enhance readability and clarity for the reader. Additionally, it is recommended to include resource consumption metrics of your developed model compared to the baseline model, such as RAM usage and training time per epoch. These details will provide a more comprehensive evaluation of the model’s efficiency. Please provide detailed visualizations of the distribution within the datasets using appropriate graphs. This will help readers better understand the data composition and support the validity of the experimental results.*

**Response：** Thank you for your question regarding the experimental configuration and dataset situation. We have added Table 3, which outlines the parameters configuration and resource consumption, and Fig 8 to illustrate the dataset's distribution.

*6. Discussion: In the Discussion section, it would be beneficial to mention potential future work, such as leveraging SAR imagery for improved performance and addressing enhancements to cope with adverse weather conditions. Including these aspects could strengthen the manuscript by highlighting promising directions for further research.*

**Response：** Thank you for your suggestion about future work in the discussion section. Indeed, adding possible future application directions and plans can provide readers with more inspiration. We have added some potential future work and application directions in the discussion section.
